# Empagliflozin in improving exercise tolerance in HFpEF: study protocol for an open-label, randomized controlled study

**DOI:** 10.1186/s13063-026-09654-y

**Published:** 2026-04-07

**Authors:** Rufeng Huang, Yecheng Deng, Mengshuang Li, Linghua Chen, Meifen Lv, Lihua Liao, Li Ma, Zhaoqi Huang

**Affiliations:** 1 Department of Cardiology, Guangdong Provincial Key Laboratory of Major Obstetric Diseases, Guangdong Provincial Clinical Research Center for Obstetrics and Gynecology, The Third Affiliated Hospital, Guangzhou Medical University, Duobao Road, Guangzhou, 510150 China; 2 Department of Geriatric Medicine, the General Hospital of Southern Theatre Command, Guangzhou, China

**Keywords:** SGLT2i, HFpEF, HFmrEF, CPET, Exercise tolerance, Study protocol

## Abstract

**Introduction:**

Heart failure with preserved ejection fraction (HFpEF) constitutes nearly half of patients with heart failure, but there is still a lack of treatment options to improve prognosis. Empagliflozin is a new hypoglycemic medication classified as sodium glucose cotransporter 2 inhibitors (SGLT2i); nonetheless, its effects on exercise tolerance in HFpEF remain ambiguous. Additional clinical studies are necessary to clarify the effect of SGLT2i in HFpEF. This study aims to evaluate the efficacy and safety of empagliflozin on exercise tolerance in patients with heart failure with preserved ejection fraction without diabetes, using cardiopulmonary exercise testing.

**Methods and analysis:**

This study is a single-center, open-label, randomized controlled trial. We will randomly (1:1) assign 86 patients with an ejection fraction of more than 40% and class II–III heart failure to receive empagliflozin (10 mg once daily) or to maintain their original treatment regimen. The treatment duration will be 12 weeks. The primary endpoint is the evaluation of changes in peak oxygen uptake (peak VO2) after a 12-week period using cardiopulmonary exercise testing (CPET). The secondary endpoints encompassed additional parameters of CPET and echocardiography, N-terminal pro-B-type natriuretic peptide level, alanine aminotransferase level, aspartate transaminase level, estimated glomerular filtration rate level, New York Heart Association functional classification, and scores from the Minnesota Living with Heart Failure Questionnaire. Safety events associated with empagliflozin and CPET will be monitored.

**Discussion:**

We used peak oxygen uptake, the gold standard for assessing exercise tolerance, to evaluate the efficacy and safety of empagliflozin in treating non-diabetic patients with HFpEF. The improvement in quality of life of heart failure patients by SGLT2i will be objectively assessed.

**Trial registration:**

www.chictr.org.cn (ChiCTR2300072908). Registered on 04 July 2023.

**Supplementary Information:**

The online version contains supplementary material available at 10.1186/s13063-026-09654-y.

## Background

Heart failure (HF) is a group of complex clinical syndromes characterized by abnormal structural and/or function of the heart due to a variety of causes, leading to impaired ventricular contraction and/or diastolic function. In patients with heart failure, those with heart failure with preserved ejection fraction (HFpEF) make up a large proportion. Community data on the prevalence and incidence of HFpEF indicate that approximately 50% of patients with the clinical syndrome of heart failure have a preserved ejection fraction [1]. HFpEF is a highly prevalent, complex, and heterogeneous disease. The universally accepted pathophysiology of HFpEF includes left ventricular diastolic dysfunction, pulmonary vascular dysfunction and right ventricular dysfunction [2]. Currently, HFpEF lacks effective therapies, and clinical treatment primarily focuses on symptom alleviation.

Sodium-glucose cotransporter 2 inhibitors (SGLT2i) is a rising star among antidiabetic drugs. Increasing research has confirmed that SGLT2i have effects beyond glucose lowering, providing benefits to multiple organs such as the heart and kidneys, particularly in cardiovascular events, with a predominant focus on heart failure. SGLT2i exert therapeutic effects in heart failure through multiple mechanisms, including the inhibition of cardiac inflammation and fibrosis, antagonism of sodium retention, and enhancement of glomerular function [3]. SGLT2i can improve myocardial energy metabolism, promote ketone production and utilization, and decelerate the progression of heart failure [4]. Consequently, SGLT2i are gradually becoming first-line medications for the treatment of heart failure.

Nonetheless, owing to the substantial disparities in the pathophysiology of HFpEF, clinical studies have failed to confirm that ACEI/ARB and beta-blockers can improve the prognosis or reduce the mortality rate of HFpEF patients. Consequently, there is a lack of evidence-based medical evidence for the treatment of HFpEF. But increasing clinical studies have found that SGLT2i can confer cardiovascular benefits to patients with HFpEF. The renowned randomized trials, EMPEROR-Preserved trial and DELIVER trial, have demonstrated that SGLT2i empagliflozin and dapagliflozin can reduce the composite risk of cardiovascular death or heart failure hospitalization in patients with HFrEF and HFpEF [5, 6]. A meta-analysis revealed that the concurrent use of ARNI, MRA, and SGLT2i exerts potent anti-heart failure effects [7]. SGLT2i in the growing role of HfpEF. Further clinical studies are still needed to confirm the role of SGLT2i in HFpEF.

Exercise intolerance is a symptom of HF, which is in connection with an increasing risk of death. Cardiopulmonary exercise testing (CPET) a diagnostic examination that assesses cardiopulmonary function. It involves a series of assessments conducted by measuring changes in cardiopulmonary function and related parameters during exercise. In individuals with heart disease, CPET can quantify the extent to which exercise limitations are attributable to cardiac factors. Peak oxygen uptake (peak VO2) is considered the foremost standard for evaluating exercise intolerance in individuals with HF [8]. Peak VO2 is also an important predictor of mortality in patients with HFpEF [9]. The parameters of peak VO2, percent predicted peak VO2,and exercise duration in CPET had the strongest ability to predict mortality [9].

We designed the study to evaluate the efficacy of empagliflozin on exercise tolerance in patients with HFpEF.

## Methods and design

### Design

**Fig. 1:**
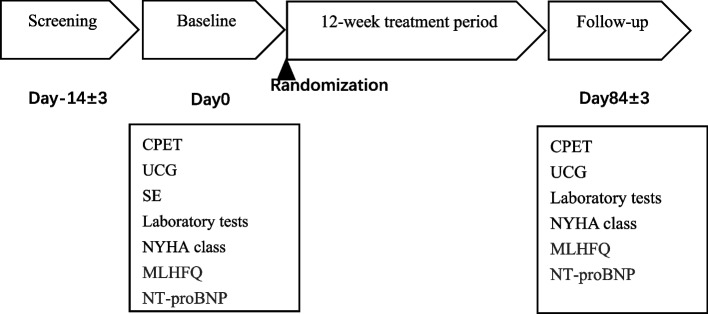
Study design ECG, electrocardiogram; UCG, ultrasound cardiogram; CPET, cardiopulmonary exercise testing; Laboratory tests: complete blood count, serum lipids and biochemical tests. MLHFQ, Minnesota Heart Failure Quality of Life Scale

This study is designed as a single-center, open-label, randomized controlled trial. After the screening period, eligible patients are randomly assigned (1:1 randomization) to receive either empagliflozin, 10 mg per day, or to continue with the original treatment plan. Figure 1 illustrates the design and procedures through the trial.

### Study hypothesis

Empagliflozin can improve exercise tolerance of patients with HFpEF.

### Study population, eligibility criteria, and recruitment

Participants are men or women aged 18 to 75 years, who have New York Heart Association (NYHA)functional class II–III chronic heart failure and a left ventricular ejection fraction of more than 40% (encompassing both HFmrEF and HFpEF, as in the EMPEROR-Preserved trial). The protocol requires patients to have an N-terminal pro–B-type natriuretic peptide (NT-proBNP) level of more than 300 pg per milliliter and estimated glomerular filtration rate (eGFR) more than 30 ml/min/1.73 m^2^.

The study primarily excludes patients with diabetes, severe liver or kidney dysfunction, as well as those with other conditions deemed unsuitable for inclusion. The key inclusion and exclusion criteria are listed in Table 1.

**Table 1:** Inclusion and exclusion criteria

**Inclusion criteria**
(1) Aged 18 to 75 years, male or female;
(2) Patients with chronic HF (NYHA class II and III) diagnosed for at least 3 months and currently in HF NYHA class II-III;
(3) LVEF>40%；
(4) NT-proBNP >300 pg/mL;
(5) eGFR≥30ml/min/1.73m^2;
(6) Able to understand and voluntarily sign the informed consent form;
**Exclusion criteria**
(1) Diabetes;
(2) Acute coronary syndrome whihin the last 2 months or planned coronary revascularization during the trial;
(3) Atrial fibrillation, cardiovascular and cerebrovascular events related to atrial fibrillation in the last 3 months;
(4) Hepatic dysfunction, defined by serum levels of transaminases more than three times the upper limit of normal at screening;
(5) Using SGLT2i within 4 weeks prior to randomization;
(6) Diseases that affect the results of CPET include arthropathy, peripheral vascular disease, lung disease and other diseases;
(7) The subject's resting ECG has the following abnormalities: (a) ventricular pre-excitation syndrome; (b) ventricular pacing rhythm; (c) resting ST segment depression of more than 2 mm; (d) left bundle branch block or any intraventricular conduction delay with QRS duration greater than 120 ms；
(8) Congenital heart defects, progressive decompensated congestive heart failure, severe valvular disease, hypertrophic obstructive cardiomyopathy, severe uncontrolled hypertension (sitting systolic blood pressure >180mmHg or diastolic blood pressure >110mmHg), severe anemia, suspected or known arterial dissection, acute myocarditis or pericardial effusion, active endocarditis, thrombophlebitis, or pulmonary embolism.
(9) History of bleeding tendency, cerebral hemorrhage, or epilepsy requiring anticonvulsant medication.
(10) Pregnant woman or breastfeeding woman.
(11) Participated in any other trials or use of any investigational drugs within the 30 days prior to enrollment.
(12) Drug abuse. The patient has a history of alcohol abuse or known drug dependence in the past 2 years.
**Withdrawal Criteria**
(1) Any suspected drug-related serious adverse events (e.g., allergic reactions).
(2) Recurrence of acute heart failure or sudden severe dysfunction of organs such as the liver or kidneys.
(3) Pregnancy.
(4) Major protocol deviations. After randomization, it is found that the subject does not meet the inclusion criteria of the study protocol or does not follow the requirements of the study protocol, and continuing to participate in the study may pose unacceptable risks to the subject's health.
(5) Loss to follow-up. The subject does not return to the clinic and cannot be successfully contacted. Information on attempts to contact the subject must be documented.
(6) Voluntary withdrawal. The subject (or the subject's legal representative) wishes to withdraw from the study. The reason for withdrawal should be recorded in the case report form.
(7) Study termination. The study is terminated by the sponsor, ethics committee, or regulatory authority.

*CPET *cardiopulmonary exercise testing, *ECG* electrocardiogram

The study will be conducted at The Third Affiliated Hospital of Guangzhou Medical University. Patients will be recruited by researchers describing this study in the outpatient service and inpatient departments. Posters containing study information will also be used within the hospital. Every patient will sign informed consent prior to enrollment.

### Randomization and allocation

Randomization sequences will be generated by an independent statistician using SAS 9.4, with a 1:1 allocation ratio and variable block sizes. To ensure allocation concealment, the sequence will be implemented using sequentially numbered, opaque, sealed envelopes. After a participant is enrolled, the investigator will open the next sequentially numbered envelope in the series to reveal the assignment. This trial is open-label; no blinding will be used after assignment.

### Blinding

This trial is not blinded. We currently lack the conditions to produce placebos.

### Intervention

Patients in the empagliflozin group will receive empagliflozin (Boehringer Ingelheim Pharma GmbH & Co. KG) 10 mg once daily in addition to the standard medication regimen. The control group will continue with the original treatment regimen. All patients will be treated for 12 weeks.

### Concomitant medication

All patients will receive treatment for HFpEF according to the authoritative guidelines on heart failure therapy released by the ESC in 2021 [10]. At the same time, patients will feel the need to take other medications, which will be recorded and analyzed.

### Outcomes

#### Primary outcome

The primary outcome is the variation in peak VO_2_ during the study period.

#### Secondary outcomes

Secondary outcomes include the following: (a) Changes in peak VO2/HR, anaerobic threshold (AT), and VE/VCO2 evaluated by cardiopulmonary exercise testing (CPET); (b) variations in left ventricular ejection fraction (LVEF), left ventricular end-diastolic volume (LVEDV), left atrial volume (LAV), E/E’, and pulmonary artery pressure (PAP) as evaluated by echocardiography; (c) changes from baseline in levels of alanine aminotransferase (ALT), aspartate aminotransferase (AST), estimated glomerular filtration rate (eGFR), and N-terminal pro-B-type natriuretic peptide (NT-proBNP) at week 12; (d) changes from baseline in New York Heart Association (NYHA) functional class and Minnesota Heart Failure Quality of Life Questionnaire (MLHFQ) scores at week 12 (see Table 2).

**Table 2:** Primary and secondary endpoints

**Primary endpoint**
The variation in peak VO_2_ during the study period.
**Secondary endpoint**
The changes of the following parameters from baseline to week 12
1. Other CPET parameters
- peak VO_2_/HR
- AT
- VE/VCO_2_
2. Ultrasound cardiogram
- LVEF
- LVEDV
-LAV
- E/E ’
- PAP
3. New York Heart Association functional class
4. Minnesota Heart Failure Quality of Life Scale.
5. NT-proBNP.
6.ALT/AST/eGFR
**Safety endpoints**
Hypoglycemia,hypotension, deterioration of kidney function, urinary tract infection, elevated LDL cholesterol.

*CPET *cardiopulmonary exercise testing

### Study timelines

#### Screening visit (Day −14 ± 3)

The timeline and inspection items are listed in Table 3. At the screening visit, participants will receive ECG, eligibility laboratory tests, vital signs measurement, and drug history registration. Laboratory data will include urine pregnancy testing, complete blood count, biochemical indexes, NT-proBNP.

**Table 3:** Schedule of assessments

Timelines	Screening Visit	Baseline Visit	Follow-Up Visit
Week	−2	0	12
Day	−14±3	0	84±3
Informed consent	✓	X	X
Inclusion/exclusion criteria	✓	✓	X
Concomitant medication	✓	✓	✓
Vital signs	✓	✓	✓
Physical examination	✓	✓	✓
urine pregnancy testing^a^	✓	X	X
12-lead ECG	✓	X	✓
CPET	X	✓	✓
Cardiac ultrasound	X	✓	✓
Laboratory tests^b^	X	✓	✓
New York Heart Association functional class	X	✓	✓
Minnesota Heart Failure Quality of Life Scale	X	✓	✓
Medical history	✓	✓	X
Adverse events^c^	✓	✓	✓

^a^Urine pregnancy testing for women of reproductive age

^b^Laboratory tests include: complete blood count, eGFR, ALT, AST, NT-proBNP

^c^Adverse events include: urinary system, hypoglycemia, renal function damage, etc

✓ indicates that the item must be completed at that time

X indicates that the item is not required at that time

#### Baseline visit (Day 0)

After completion of baseline data collection, participants who meet the inclusion criteria and do not meet any exclusion criteria will be enrolled in the trial. Participants will be randomly assigned, in a 1:1 ratio, to either empagliflozin or continue with the original treatment plan. Subjects will complete all baseline examinations, including CPET, ultrasound cardiogram, New York Heart Association functional class and MLHFQ scores. Following baseline data collection, participants assigned to the empagliflozin group will receive empagliflozin 10 mg once daily in addition to their original treatment, while those in the control group will continue with their original treatment regimen.

#### Follow-up visit (Day 84 ± 3)

We will conduct repeat assessments of all examinations including CPET and echocardiography to evaluate efficacy and safety.

### Data collection and analysis

#### Sample collection and laboratory measurements

Blood samples will be collected during the screening visit and follow-up visits by professional nurses in The Third Affiliated Hospital of Guangzhou Medical University and measure blood routine examination, biochemical indices, NT-proBNP in clinical lab. Women of reproductive age will be asked to provide urine samples for a pregnancy test during the screening visit.

#### Cardiopulmonary exercise testing

CPET will be conducted by professional physicians in the cardiac function department. Prior to the test, physicians will thoroughly review the patient’s medical history and medication history exclude any contraindications and obtain signed informed consent for the trial. CPET consists of two parts, static pulmonary function testing and the ramp protocol. Static pulmonary function testing will obtain respiratory parameters such as forced vital capacity and maximal flow-volume loops. The ramp protocol includes several stages: a rest period, an unloaded warm-up period, a power load period, and a recovery period. The power increment for the ramp protocol is calculated using the following formula: Predicted unloaded VO_2_ (ml·min^−1^) = 150 + [6 × weight (kg)]; Predicted peak VO_2_ (ml·min^−1^) = [height (cm) - age (years)] × 20 (males) or × 14 (females) [11]. Finally, parameters such as peak VO_2_, AT, and VE/VCO_2_ are obtained. CPET reports will be issued by professional physicians and reviewed by senior physicians.

#### Cardiac ultrasound

Echocardiography is performed by professional physicians. It primarily measures indices reflecting left ventricular systolic and diastolic function, including LVEF, LVEDV, LAV, E/E', and PAP. The modified Simpson’s method is utilized for LVEF measurement, tissue Doppler imaging is employed to measure E and e′ values to derive E/e′, and PAP is assessed. Three-dimensional color Doppler echocardiography is used for LAV measurement.

### Adverse events and safety

Adverse events are categorized into two aspects: CPET-related adverse events and empagliflozin-related adverse events. Empagliflozin-related most common adverse reactions include hypoglycemia, hypotension, deterioration of kidney function, urinary tract infection, elevated LDL cholesterol levels will be monitored. According to the results from Phase III clinical trials, empagliflozin is generally well tolerated, with a low incidence of adverse events (2.2% in empagliflozin 10 mg) [12]. Additionally, common adverse reactions are generally of mild intensity, rarely led to drug discontinuation, and tended not to recur. All adverse events will be documented in the case report form for evaluation and reporting to the Clinical Research and Applied Ethics Committee of The Third Affiliated Hospital of Guangzhou Medical University.

### Data management and monitoring

After each follow-up visit, research data will be collected and documented in the case report form. All data will be kept by the investigators. Upon completion of the study, the data will be entered into the Clinical Trial Management Public Platform (medresman.org.cn) for data sharing. All data will be preserved permanently.

No interim analysis is planned for this trial due to the short study duration, moderate sample size, and the well-established safety profile of empagliflozin. All efficacy and safety outcomes will be analyzed and reported collectively upon completion of data collection.

### Data protected

All electronic participant data will be stored permanently on a hospital-standard encrypted server. Access is strictly limited to authorized research personnel directly involved in the study. All study staff receive mandatory training on data confidentiality covering relevant regulations and institutional policies. Physical documents containing participant identifiers are kept in locked cabinets within access-controlled rooms.

### Compliance evaluation

The study period is 3 months, during which subjects are required to attend monthly clinic follow-ups. Compliance will be measured using a combination of methods to enhance reliability: (1) the number of completed follow-up visits, (2) pill count based on returned medication bottles, and (3) verification of medication usage during each clinic visit. In addition, regular telephone follow-ups will be conducted to reinforce adherence and remind subjects to take their medication as scheduled.

### Strategies to enhance adherence

To improve participants’ adherence, we will (1) have our professional physicians conduct regular follow-ups, (2) provide all examinations free of charge during the trial, and (3) offer a modest compensation upon completion. These details have been added to the revised manuscript.

### Statistical consideration

#### Sample size assumptions

This study adopted a one-to-one parallel design, evaluating the sample size based on the change in peak oxygen uptake before and after treatment as the primary efficacy endpoint. It was designed as a superiority trial to compare the means of two groups. Based on previous studies, the increase in peak oxygen uptake after 6 months of treatment was estimated to be 1.1 ± 2.6 ml/kg/min for the empagliflozin group and −0.5 ± 1.9 ml/kg/min for the control group [13]. It was assumed that a difference greater than 0 between the test and control groups would be clinically significant. The combined standard deviation of the change values for both groups was set at 2.4 ml/kg/min. The sample size was calculated using the following formula:

$$N_c=\frac{\left(Z_{1-\alpha}+Z_{1-\beta}\right)^2\;\sigma^2\;\left(1+{\displaystyle\frac1r}\right)}{\left(\mu_E-\mu_c-\triangle\right)^2}$$

where *N*: required sample size per group; Z_1−α/2_: critical *Z*-value for the two-sided significance level (α); *Z*_1−β_: critical *Z*-value for the desired statistical power (1-β); *σ* pooled standard deviation of the change in peak VO₂; μ_E_: mean change in peak VO₂ for the empagliflozin group; μ_C_: mean change in peak VO₂ for the control group; Δ: the non-inferiority or superiority margin. Here, Δ = 0 defines the test for superiority; *r*: allocation ratio (n_E_/n_C_).

In this study, *α* = 0.0 25, *β* = 0.2, Z_1−α_ = 1.96, *Z*_1−β_ = 0.8416, μ E - μ C = 1.6, *σ* = 2.4, *r* = 1, Δ = 0. Calculations show that 36 subjects are required in each group. Consequently, a total of 86 subjects (43 individuals in each group) are included, accounting for an approximate dropout rate of 20%.

### Statistical analysis

Statistical analysis will be conducted using SAS 9.4. All statistical tests will be two-tailed, and a *p*-value of less than 0.05 will be considered statistically significant, unless otherwise specified.

For continuous data, statistical descriptions will include counts, means, standard deviations, medians, minimum and maximum values, and upper and lower quartiles. For categorical data, counts and percentages will be used for statistical descriptions. Between-group comparisons of continuous data will be conducted using *t*-tests or rank-sum tests, while categorical data will be compared using chi-square tests or Fisher’s exact test. Non-parametric methods, such as the Wilcoxon rank-sum test, will be employed for ordinal data. Continuous data will be presented as mean ± standard deviation (x ± s). Normality tests will be conducted first, and if both groups meet the assumptions of normality and have equal variances, a *t*-test will be used for between-group comparisons. Otherwise, we will consider the non-parametric Wilcoxon rank-sum test.

Missing data for the primary efficacy endpoint will be addressed using multiple imputation under the assumption of missing at random. Twenty imputed datasets will be generated using predictive mean matching incorporating baseline characteristics. The pooled estimates from the imputed datasets will be used for the primary analysis. Additionally, a complete-case analysis will be conducted as a validation to evaluate the robustness of the results.

#### Primary efficacy endpoint analysis

The primary efficacy endpoint, the change in peak VO₂ from baseline to week 12, will be analyzed using an analysis of covariance model. Additionally, we will compute the one-sided 97.5% confidence interval for the difference in changes in peak oxygen uptake before and after treatment, comparing both the test and control groups. If the lower boundary of this one-sided 97.5% confidence interval exceed 0, we will conclude that the test group demonstrates superiority over the control group. Furthermore, exploratory multivariate models will be applied to adjust for other potential baseline confounders.

## Discussion

The characteristic of HFpEF is impaired diastolic function, especially in elderly heart failure patients who tend to adopt a sedentary lifestyle, leading to reduced cardiopulmonary exercise tolerance and diminished quality of life. Exercise intolerance is a hallmark symptom of heart failure. SGLT2i have been shown to provide significant benefits for heart failure patients, as evidenced by the Emperor-Preserved study. While the majority of previous studies on SGLT2i in heart failure have primarily focused on hospitalization and mortality rates, there has been limited research on patients’ exercise tolerance. In response to the issue of decreased exercise tolerance in HFpEF, we conducted a more in-depth investigation. The efficacy of empagliflozin on exercise tolerance was assessed through cardiopulmonary exercise testing, evaluating improvements in both exercise tolerance and cardiac function.

Exercise intolerance is a hallmark symptom of heart failure, associated with increased disability and mortality risk. Although previous studies have demonstrated that SGLT2i improves the outcome of the six-minute walk test in patients with HFpEF [14]. However, compared with the six-minute walk test, the results of the CPET are more accurate and objective. Cardiopulmonary exercise testing evaluates a series of cardiopulmonary functions by measuring changes in relevant parameters during exercise. Furthermore, Peak VO₂ and a range of CPET indicators serve as strong evidence for assessing prognosis in heart failure patients [15]. Our study selected peak oxygen uptake as the primary endpoint because it is considered the gold standard for assessing exercise tolerance. A study compared the results of CPET and tissue Doppler in 32 HFpEF patients, revealing a significant correlation between the impairment of peak VO_2_ and the impairment of left ventricular filling pressure parameters, indirectly demonstrating an association between peak VO_2_ with impaired left ventricular diastolic function [16]. Additionally, our research incorporated the MLHFQ and New York Heart Association functional classification to subjectively assess patients’ quality of life, aiming to obtain the most authentic results through a comprehensive evaluation. Our goal is to demonstrate that empagliflozin improves exercise tolerance and living quality in patients with HFpEF. This could further support the therapeutic role of SGLT2i in HFpEF.

Additionally, in previous studies, the intervention period for SGLT2i in HFpEF patients has typically been over 6 months. Short-term effects of empagliflozin have not been explored. Our study has an intervention period of 3 months to observe whether empagliflozin has short-term therapeutic effects on HfpEF.

Currently, studies have confirmed the efficacy of SGLT2i in the treatment of HFpEF. Our research has observed the therapeutic response of non-diabetic patient populations to empagliflozin. However, several issues remain unresolved regarding the treatment of HFpEF with empagliflozin. Firstly, while there is some data on the efficacy of empagliflozin as a monotherapy, further research is needed to investigate its effects and potential interactions when used in combination with other HFpEF treatment medications. This is crucial for determining the optimal therapeutic regimen. A recent meta-study showed that in patients with HF and LVEF>40%, the quadruple combination of ARNI, BB, MRA, and SGLT2i provides the largest reduction in the risk of Cardiovascular death [7]. But there is a lack of real-world data. The clinical trial environment and real-world clinical practice settings differ significantly. More real-world data from everyday clinical practice is needed to assess the actual application. Building upon the findings of this study and the unresolved issues outlined above, we plan to conduct a subsequent multicenter, prospective, observational real-world study. This study aims to enroll a broader spectrum of HFpEF patients and follow them over an extended period in routine clinical practice settings, where empagliflozin is used either as monotherapy or in combination with other agents. We will systematically collect data on drug persistence, dose adjustments, combination therapy patterns, clinical outcomes and patient-reported outcomes. We anticipate that this future research will provide more robust evidence regarding the real-world effectiveness and safety of empagliflozin and help optimize individualized treatment strategies for HFpEF.

### Trial status

The protocol version number is 2.0, with the version date being April 20, 2022. This study was registered at www.chictr.org.cn on July 4, 2023. However, due to a delay in receiving funding, the actual recruitment start date was pushed to January 2024. Considering the limited number of patients currently meeting the inclusion criteria, the study remains in the patient enrollment phase, with recruitment anticipated to be completed by the end of 2025.

## Supplementary Information

Additional file 1: SPIRIT Checklist.

## Abbreviations

CPET: Cardiopulmonary exercise testing
eGFR: Estimated glomerular filtration rate
HF: Heart failure
HFpEF: Heart failure with preserved ejection fraction
NYHA: New York Heart Association
NT-proBNP: N-terminal pro–B-type natriuretic peptide
peak VO2: Peak oxygen uptake
SGLT2i: Sodium-glucose cotransporter 2 inhibitors
